# Schinzel-Giedion Syndrome in One of Dizygotic Twins: Confirmation of a De Novo SET Binding Protein 1 (SETBP1) Variant and Classic Multisystem Phenotype

**DOI:** 10.7759/cureus.108270

**Published:** 2026-05-04

**Authors:** Sanjay Reddy Thanugundla, Sri Lakshmi Kothakapa, Sulekha Ramireddy, Sainithya Chittireddy, Parimi Vamsi Krishna

**Affiliations:** 1 General Medicine, Malla Reddy Institute of Medical Sciences, Hyderabad, IND; 2 Internal Medicine, Malla Reddy Institute of Medical Sciences, Hyderabad, IND; 3 Internal Medicine, J.J.M. Medical College, Davangere, IND

**Keywords:** craniofacial dysmorphism, developmental delay, dizygotic twins, hydronephrosis, schinzel-giedion syndrome, setbp1

## Abstract

Schinzel-Giedion syndrome (SGS) is a very rare congenital disorder linked to de novo pathogenic variants in the SET Binding Protein 1 (SETBP1) gene. The condition is usually suspected from a recognisable craniofacial pattern, severe developmental delay, early neurological problems, and congenital abnormalities involving several organ systems. As the disorder is uncommon and presents with wide systemic involvement, diagnosis can be missed or delayed unless the clinical features are carefully recognised and followed by appropriate genetic testing.

We report the case of a two-year-old girl who was delivered at 33 weeks and two days as the first-born infant in a dizygotic twin pregnancy. She showed a recognisable craniofacial phenotype along with severe developmental delay, hypotonia, early-onset seizures, progressive hydrocephalus, marked bilateral hydronephrosis with vesicoureteral reflux, recurrent urinary tract infections, a large secundum atrial septal defect, recurrent respiratory illness, congenital talipes equinovarus, and bilateral sensorineural hearing loss. Molecular analysis detected a pathogenic de novo SETBP1 variant, which confirmed the diagnosis of SGS. A heterozygous Glutaredoxin and Cysteine-Rich Domain Containing 1 (GRXCR1) variant of uncertain significance (VUS) was also identified, but it was not considered sufficient to account for the hearing loss, as GRXCR1-related hearing impairment is typically autosomal recessive and requires stronger supporting evidence. Her male co-twin was clinically normal and had no dysmorphic features, favouring a sporadic event in this twin pregnancy.

This case underlines the importance of craniofacial dysmorphism as an early clue to SGS in a child with multisystem disease. It also shows why molecular results must be interpreted cautiously, especially when variants of uncertain significance are detected. Early genetic confirmation, counselling of the family, and coordinated care by paediatric neurology, nephrology, cardiology, genetics, and rehabilitation teams are essential for appropriate long-term management.

## Introduction

Schinzel-Giedion syndrome (SGS) is a rare congenital malformation disorder caused by pathogenic variants in the SET binding protein 1 (SETBP1) gene. It is characterised by a recognisable craniofacial phenotype, severe developmental delay, neurological involvement, and congenital abnormalities affecting multiple systems, particularly the renal, cardiac, skeletal, and respiratory systems [1-6]. Although the facial appearance may suggest the diagnosis in well-evolved cases, early recognition can be difficult because infants may initially present with non-specific or organ-specific problems such as feeding difficulty, seizures, hydronephrosis, recurrent infections, or poor growth [2-6].

The molecular basis of SGS was clarified after the identification of de novo gain-of-function variants in SETBP1 [7]. Many pathogenic variants cluster within the degron region of the protein, resulting in impaired protein degradation and abnormal protein accumulation [7-9]. This genotype-phenotype association supports the role of SETBP1 in the classic SGS phenotype; however, genetic findings must always be interpreted in relation to the clinical presentation, as different classes of SETBP1 variants may be associated with different disorders [7-10].

The clinical spectrum of SGS includes craniofacial dysmorphism, profound developmental delay, hypotonia, seizures, structural brain abnormalities, hydronephrosis, vesicoureteral reflux, congenital heart defects, and skeletal deformities such as talipes equinovarus [3-6,11-15]. Because of this multisystem involvement, diagnosis depends on careful phenotyping supported by molecular confirmation. Early diagnosis helps in family counselling, prognostic discussion, surveillance for complications, and planning multidisciplinary care [13-16].

Molecular results should also be interpreted cautiously when additional variants are detected. For example, glutaredoxin and cysteine-rich domain containing 1 (GRXCR1)-related nonsyndromic hearing loss is usually autosomal recessive; therefore, a single heterozygous GRXCR1 variant of uncertain significance (VUS) should not be considered causative without a second pathogenic variant in trans or other supportive evidence [16,17]. This is particularly important in children with complex syndromic presentations, where incidental or uncertain variants may be identified during broad genetic testing.

The present case highlights the diagnostic value of craniofacial recognition in a child with severe multisystem involvement. It also illustrates the need to combine clinical examination, imaging, audiological evaluation, cardiac assessment, and molecular testing for accurate diagnosis and coordinated long-term management in SGS.

## Case presentation

A two-year-old girl was admitted with respiratory distress, poor oral intake, and decreased urine output. According to the caregivers, she had passed urine only once during the preceding 24 hours. There was no history of fever, coryza, or dysuria at the time of presentation. On admission, she was noted to have increased work of breathing, tachypnoea, and reduced activity. The presenting clinical features are summarised in Table 1.

**Table 1:** Key clinical features ENT: Ear, nose, and throat.

System	Findings
Craniofacial	Prominent forehead, hypertelorism, midface hypoplasia, bitemporal narrowing, wide fontanelles, protruded tongue, low-set ears
Dermatological	Forehead hypertrichosis
Neurological	Severe developmental delay, hypotonia, early-onset seizures, progressive hydrocephalus
Respiratory	Recurrent pneumonia, bronchiolitis, tachypnea
Renal	Bilateral grade 4 hydronephrosis, vesicoureteral reflux, recurrent urinary tract infection
Cardiac	Large secundum atrial septal defect with right heart dilatation
Auditory / ENT	Bilateral sensorineural hearing loss, oral candidiasis
Musculoskeletal	Left congenital talipes equinovarus
Other	Feeding difficulty; lumbar soft tissue lesion

She was the first-born of dizygotic twins delivered by lower-segment caesarean section at 33 weeks and two days of gestation for spontaneous preterm labour. The pregnancy was non-consanguineous. Maternal history was significant for hypothyroidism and polycystic ovarian syndrome. Antenatal ultrasonography had demonstrated polyhydramnios. There was no family history of a similar congenital or neurodevelopmental disorder. Her male co-twin was clinically normal and did not have dysmorphic features.

**Figure 1:**
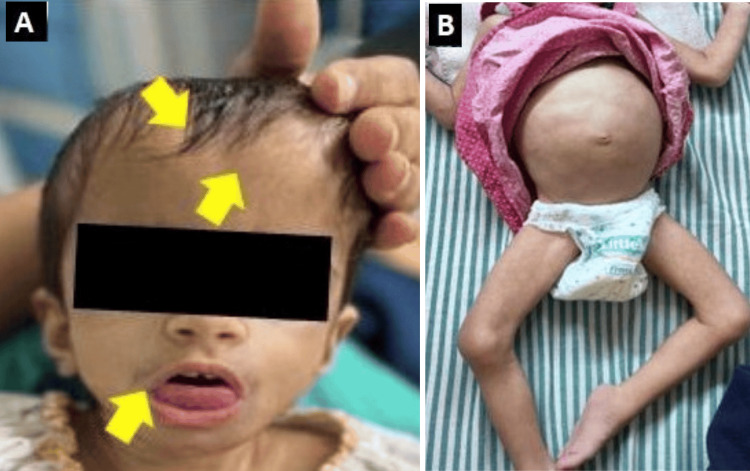
Clinical photographs of the child showing the characteristic external phenotype (A) Frontal facial view demonstrating dysmorphic craniofacial features, including frontal prominence, midface hypoplasia, and protruding tongue; the arrows highlight the abnormal craniofacial landmarks; (B) Whole-body view from the neck down showing marked growth failure, abdominal distension, reduced subcutaneous tissue, and abnormal lower-limb posturing.

At birth, the child weighed 1.4 kg, with a length of 40 cm and head circumference of 30 cm. Neonatal records documented hypotonia and reduced spontaneous movements. At two years of age, physical examination showed a distinct dysmorphic phenotype characterised by prominent forehead, hypertelorism, bitemporal narrowing, midface hypoplasia, depressed nasal bridge, low-set ears, protruding tongue, wide fontanelles, and frontal hypertrichosis. These craniofacial features are demonstrated in the clinical photographs in Figure 1, showing prominent forehead, hypertelorism, depressed nasal bridge, protruding tongue, and frontal hypertrichosis (Figure 1).

**Figure 2:**
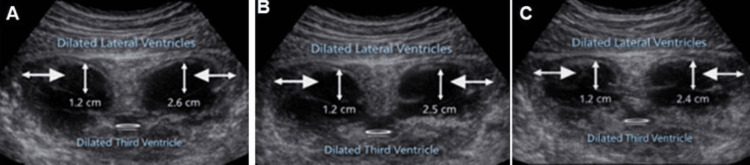
Neurosonographic images demonstrating mild hydrocephalus The three panels show cranial ultrasound sections with enlargement of the lateral ventricles and mild dilatation of the third ventricle. The measured ventricular dimensions are displayed within each image (A, B, C) illustrating persistent ventriculomegaly across the examined views.

Neurologically, the child had severe global developmental delay, generalized hypotonia, and early-onset seizures. Cranial imaging documented progressive ventricular dilatation. Neurosonography performed at four months showed mild hydrocephalus with dilated lateral ventricles, and repeat neurosonography at two years demonstrated worsening hydrocephalus with ventriculomegaly and diffuse hyperechoic foci, supporting progressive central nervous system involvement (Figure 2).

**Figure 3:**
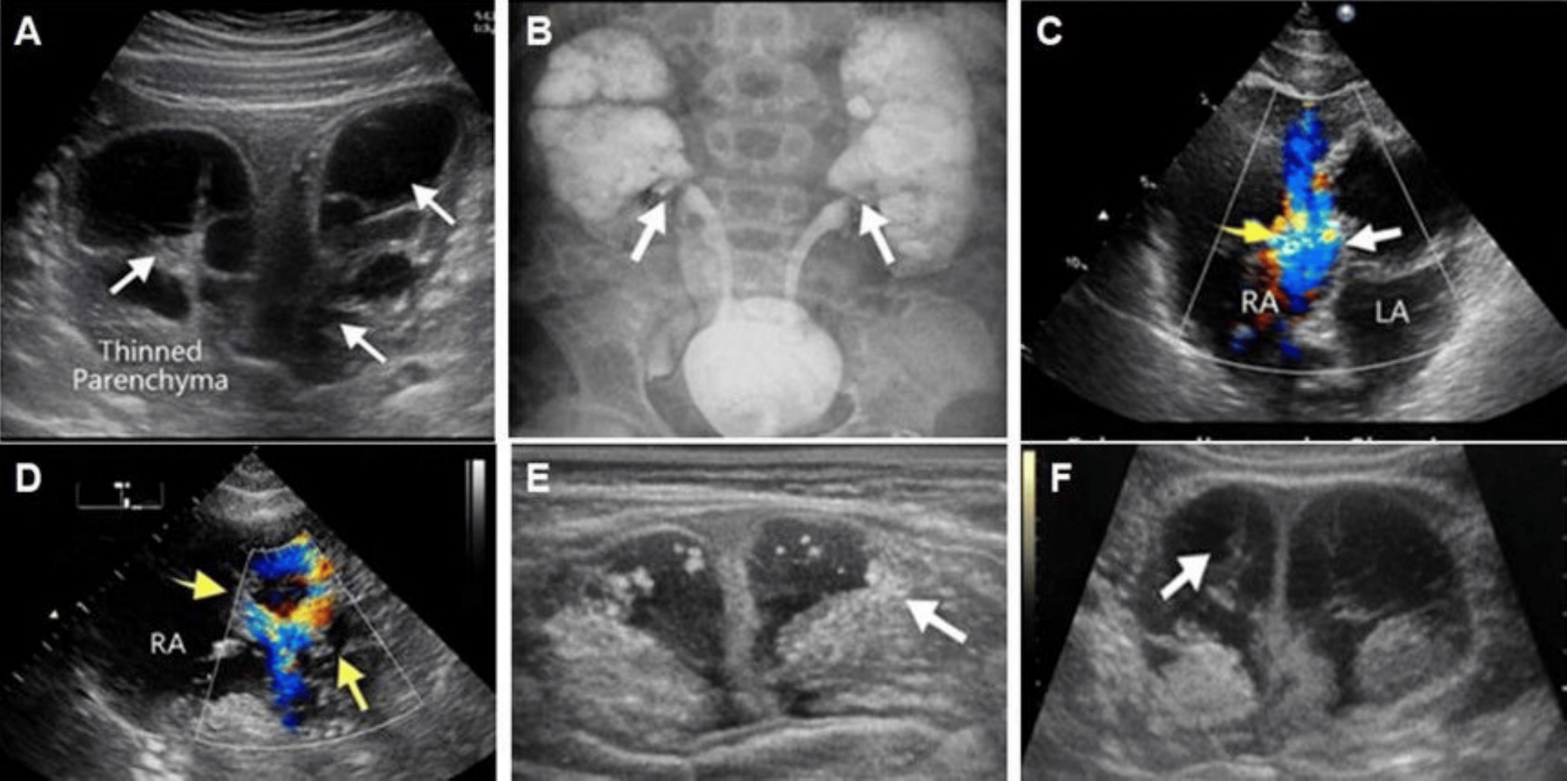
Imaging studies A) Bilateral grade IV hydronephrosis with parenchymal thinning; B) VCG/MCUG showing bilateral vesicoureteral Reflux; C, D) Echocardiography showing large secundum ASD with moderate right heart dilatation; D) Echocardiography showing large secundum ASD with moderate right heart dilatation; E, F) USG showing lumbar soft tissue lesion. USG: Ultrasonography; ASD: Atrial septal defect; VCG/MCUG: Voiding cystography / micturating cystourethrography

Figure 3 demonstrates significant renal and cardiac involvement in this child.

The renal abnormalities are shown in Figure 3A and Figure 3B.

The ultrasound image in Figure 3A shows marked bilateral hydronephrosis with dilatation of the pelvicalyceal systems and associated thinning of the renal parenchyma, suggesting advanced upper urinary tract damage. The contrast study in Figure 3B demonstrates bilateral vesicoureteral reflux, with reflux of contrast from the urinary bladder into both ureters and renal collecting systems. Taken together, these renal findings indicate severe structural urinary tract disease (Figures 3A, 3B).

The cardiac abnormality is illustrated in Figures 3C, 3D. These echocardiographic images show a large secundum atrial septal defect (ASD) with abnormal colour flow across the atrial septum. The appearance is also consistent with dilatation of the right-sided cardiac chambers, indicating haemodynamic burden from left-to-right shunting (Figures 3C, 3D).

The remaining panels further support the multisystem nature of the disease. Figures 3E, 3F depict the lumbar soft tissue lesion on ultrasound, shown as a well-defined soft tissue abnormality in the lumbosacral region. Overall, the figure documents severe renal pathology along with significant congenital cardiac disease, in keeping with the broad systemic involvement seen in this syndrome (Figures 3A-3F). 

Additional systemic findings further supported the multisystem nature of the child’s illness. She had bilateral sensorineural hearing loss, recurrent oral candidiasis, feeding difficulty, and left-sided congenital talipes equinovarus. A soft tissue swelling was also noted in the lumbar region during clinical evaluation. Imaging of the lumbosacral area showed a well-defined soft tissue lesion measuring approximately 3 × 3 cm. The lesion was documented in relation to its lumbar location, depth from the skin surface, and internal echotexture, with no clear evidence of spinal canal communication.

Molecular confirmation was obtained when the child was three months old. Clinical exome sequencing detected a heterozygous likely pathogenic missense variant in the SETBP1 gene, c.2612T>C (p.Ile871Thr), located in exon 4, supporting the diagnosis of SGS (OMIM #269150). This molecular finding was consistent with the child’s clinical presentation, including craniofacial dysmorphism, severe developmental delay, neurological involvement, renal abnormalities, cardiac defect, and skeletal features. An additional heterozygous VUS was identified in GRXCR1, c.545G>A (p.Gly182Glu), transcript ENST00000399770.3. As GRXCR1-related nonsyndromic hearing loss is usually inherited in an autosomal recessive pattern, a single heterozygous VUS was not considered sufficient to explain the child’s sensorineural hearing loss [17] (Table 2).

**Table 2:** Genetic and radiological investigations

Investigation	Finding
SETBP1 gene testing	Pathogenic de novo mutation confirming Schinzel-Giedion syndrome
GRXCR1 gene testing	Mutation associated with autosomal recessive non-syndromic hearing loss
Neurosonogram, 4 months	Mild hydrocephalus with dilated lateral ventricles
Neurosonogram, 2 years	Worsening hydrocephalus / ventriculomegaly with diffuse hyperechoic foci
2D echocardiography, 3 months	Large secundum atrial septal defect with moderate right heart dilatation
Ultrasound abdomen, neonatal period	Bilateral grade 4 hydronephrosis with increased renal echogenicity
Ultrasound abdomen, 2 years	Pelvicalyceal dilatation with parenchymal thinning, bladder debris, overdistended gallbladder
Lumbar imaging	Soft tissue lesion approximately 3 x 3 cm

The child was managed with supportive multidisciplinary care. During admission and follow-up, treatment included oxygen supplementation, intravenous fluids, antiseizure medications consisting of levetiracetam, clobazam, and valproate, thyroid hormone replacement, diuretics, antibiotic prophylaxis, zinc supplementation, gastric protection, nutritional support, and rehabilitative interventions. Longitudinal follow-up was provided through paediatric neurology, nephrology, cardiology, clinical genetics, and developmental services

Permission for publication of the clinical details and anonymised clinical photographs was obtained from the child’s parents/legal guardian

## Discussion

This case demonstrates a classic yet severe presentation of SGS, a rare developmental disorder caused by pathogenic variants in SETBP1 [1,2]. In the present child, the diagnosis was first suspected from the characteristic craniofacial appearance and was subsequently confirmed by molecular testing. This sequence reflects an important clinical reality in rare genetic syndromes: even in the genomic era, careful phenotypic assessment often provides the first meaningful clue to diagnosis [2-5]. The combination of prominent forehead, hypertelorism, midface hypoplasia, bitemporal narrowing, wide fontanelles, low-set ears, and hypertrichosis created a recognisable facial pattern that strongly pointed toward SGS early in the evaluation [2,5].

The patient also exhibited the multisystem involvement that defines this syndrome. Neurological manifestations included severe global developmental delay, hypotonia, seizures, and progressive hydrocephalus. These findings are in keeping with earlier descriptions of SGS, in which neurological impairment is one of the most consistent and disabling components of the disease [3-6]. Progressive ventriculomegaly, developmental regression or stagnation, and refractory seizures have all been reported, reflecting the significant central nervous system burden associated with this condition [2-6]. In the present case, the severity of the neurological abnormalities underscored the aggressive clinical course and the need for close long-term surveillance.

Renal involvement was another major contributor to morbidity. The child had bilateral grade IV hydronephrosis with vesicoureteral reflux, both of which are well-recognised features of SGS [2,4,5]. Urinary tract anomalies are common in affected children and may account for recurrent urinary tract infections, worsening renal function, repeated admissions, and persistent clinical instability [2,5]. In this patient, the severity of the renal disease likely played a central role in the recurrent illnesses and overall frailty. The associated parenchymal thinning on imaging further emphasised the seriousness of the renal pathology.

Additional systemic abnormalities, including a large secundum ASD, recurrent respiratory disease, and congenital talipes equinovarus, further broadened the phenotype. These findings are consistent with the known multisystem nature of SGS and reinforce that the disorder should not be viewed as purely neurological or craniofacial [2,4,5]. Cardiac defects, skeletal deformities, and respiratory complications have all been described in previously reported cases and often add substantially to clinical complexity and care burden [2,5]. The coexistence of these abnormalities in the present child reflects the extensive organ involvement that frequently accompanies severe SGS.

Bilateral sensorineural hearing loss was present in this child as part of the clinical phenotype. Clinical exome sequencing also identified a heterozygous GRXCR1 VUS. This finding has been interpreted cautiously, as GRXCR1-related nonsyndromic hearing loss is generally inherited in an autosomal recessive pattern, and a single heterozygous VUS is not sufficient to establish causality. Therefore, the GRXCR1 variant has been recorded as an incidental uncertain molecular finding rather than an explanation for the hearing loss. This case underlines the importance of correlating additional genetic variants with inheritance pattern, clinical findings, and available evidence before assigning phenotypic relevance.

The discordant occurrence of the disorder in dizygotic twins adds further significance to this report. Only the female twin was affected, while the male co-twin remained clinically normal. This pattern strongly supports the sporadic and de novo nature of the SETBP1 mutation, which is the usual mechanism in SGS [1,2]. From a genetic counselling perspective, this is important because it demonstrates that even in a twin pregnancy, a severe monogenic disorder may occur in only one child without any contributory family history. This observation also reinforces the importance of molecular confirmation in apparently isolated cases.

There is currently no curative therapy for SGS, and management remains supportive, symptom-based, and multidisciplinary [2,5]. Nevertheless, early diagnosis has clear practical value. Prompt recognition allows structured monitoring for neurological progression, renal complications, cardiac disease, respiratory morbidity, feeding difficulty, and developmental needs. It also helps clinicians organise coordinated follow-up and enables more informed counselling of families regarding prognosis and expected complications [2,5]. In disorders with such extensive multisystem involvement, diagnostic delay may directly affect both quality of care and anticipatory management.

This case also carries an important bedside message. In any child presenting with global developmental delay and multiple congenital anomalies, careful dysmorphic examination remains highly informative [2,5,7,8]. Recognition of the characteristic craniofacial phenotype can substantially shorten the path to diagnosis and guide timely genetic testing. As shown in this patient, early clinical suspicion based on phenotype, followed by targeted molecular confirmation, remains one of the most effective approaches in rare syndromic disorders.

## Conclusions

SGS is a rare multisystem disorder in which the craniofacial pattern may provide an important early clue to diagnosis. In this child, the facial dysmorphism was associated with severe developmental delay, seizures, hydrocephalus, marked renal involvement, congenital heart disease, skeletal abnormality, recurrent respiratory illness, and hearing loss. Molecular testing identified a de novo pathogenic SETBP1 variant, confirming the diagnosis. The additional heterozygous GRXCR1 variant was recorded as a VUS and was not considered sufficient to explain the hearing loss in the absence of supportive genetic evidence. The normal phenotype of the male co-twin supports the sporadic occurrence of the disorder in this dizygotic twin pregnancy. Early clinical suspicion, appropriate molecular confirmation, genetic counselling, and coordinated multidisciplinary care are essential for managing children with this complex and life-limiting condition.

## References

[1] Hoischen A, van Bon BW, Gilissen C (2010). De novo mutations of SETBP1 cause Schinzel-Giedion syndrome. Nat Genet.

[2] Liu WL, He ZX, Li F, Ai R, Ma HW (2018). Schinzel-Giedion syndrome: a novel case, review and revised diagnostic criteria. J Genet.

[3] Pul M, Yilmaz N, Komsuoglu B (1990). The Schinzel-Giedion syndrome: a case report and review of the literature. Clin Pediatr (Phila).

[4] Takeuchi A, Okamoto N, Fujinaga S (2015). Progressive brain atrophy in Schinzel-Giedion syndrome with a SETBP1 mutation. Eur J Med Genet.

[5] Lehman AM, McFadden D, Pugash D, Sangha K, Gibson WT, Patel MS (2008). Schinzel-Giedion syndrome: report of splenopancreatic fusion and proposed diagnostic criteria. Am J Med Genet A.

[6] Al-Mudaffer M, Oley C, Price S, Hayes I, Stewart A, Hall CM, Reardon W (2008). Clinical and radiological findings in Schinzel-Giedion syndrome. Eur J Pediatr.

[7] Piazza R, Magistroni V, Redaelli S (2018). SETBP1 induces transcription of a network of development genes by acting as an epigenetic hub. Nat Commun.

[8] Acuna-Hidalgo R, Veltman JA, Hoischen A (2016). New insights into the generation and role of de novo mutations in health and disease. Genome Biol.

[9] Kelley RI, Zackai EH, Charney EB (1982). Congenital hydronephrosis, skeletal dysplasia, and severe developmental retardation: the Schinzel-Giedion syndrome. J Pediatr.

[10] al-Gazali LI, Farndon P, Burn J, Flannery DB, Davison C, Mueller RF (1990). The Schinzel-Giedion syndrome. J Med Genet.

[11] Carvalho E, Honjo R, Magalhães M (2015). Schinzel-Giedion syndrome in two Brazilian patients: report of a novel mutation in SETBP1 and literature review of the clinical features. Am J Med Genet A.

[12] Zheng J, Gu M, Xiao S, Li C, Mi H, Xu X (2024). Novel SETBP1 D874V adjacent to the degron causes canonical schinzel-giedion syndrome: a case report and review of the literature. BMC Pediatr.

[13] Herenger Y, Stoetzel C, Schaefer E (2015). Long term follow up of two independent patients with Schinzel-Giedion carrying SETBP1 mutations. Eur J Med Genet.

[14] Bulut O, Ince Z, Altunoglu U, Yildirim S, Coban A (2017). Schinzel-Giedion syndrome with congenital megacalycosis in a Turkish Patient: report of SETBP1 mutation and literature review of the clinical features. Case Rep Genet.

[15] Beaman GM, Jarvis BW, Goyal A (2025). Case Report: Prolonged survival in Schinzel-Giedion syndrome featuring megaureter and de novo SETBP1 mutation. Front Pediatr.

[16] Sullivan JA, Stong N, Baugh EH, McDonald MT, Takeuchi A, Shashi V (2020). A pathogenic variant in the SETBP1 hotspot results in a forme-fruste Schinzel-Giedion syndrome. Am J Med Genet A.

[17] Schraders M, Lee K, Oostrik J (2010). Homozygosity mapping reveals mutations of GRXCR1 as a cause of autosomal-recessive nonsyndromic hearing impairment. Am J Hum Genet.

